# The impact of Chinese undergraduates’ perceived acceptance of AI technology on academic achievement: the mediating role of critical thinking

**DOI:** 10.3389/fpsyg.2025.1727037

**Published:** 2025-12-12

**Authors:** Cheng Liu, Kexuan Zhu, Zijing Zhang, Yue Ding

**Affiliations:** 1 School of Economics and Business Management, Harbin Institute of Petroleum, Harbin, China; 2 Chinese International College, Dhurakij Pundit University, Bangkok, Thailand; 3 Development Planning Office, Guizhou University of Traditional Chinese Medicine, Guiyang, China

**Keywords:** AI technology acceptance, academic achievement, critical thinking, technology acceptance model, Chinese undergraduates

## Abstract

**Introduction:**

Although AI has become an integral part of university students’ daily learning and career development, existing research rarely examines how students’ acceptance of AI technology influences the cultivation of their critical thinking abilities. This study emphasizes the impact of Chinese undergraduates’ perceived acceptance of AI technology (perceived ease of use and perceived usefulness) on academic achievement, alongside the mediating role of critical thinking.

**Methods:**

Utilizing a questionnaire survey, this study targets undergraduate students in Chinese universities, surveying 549 participants from 4 universities in China.

**Results:**

According to our study results, perceived usefulness of AI technology significantly and positively impacts the academic achievement (*β* = 0.117, *p* < 0.050) and critical thinking (*β* = 0.332, *p* < 0.001); perceived ease of use of AI technology significantly and positively impacts the academic achievement (*β* = 0.363, *p* < 0.001) and critical thinking, separately (*β* = 0.313, *p* < 0.001); critical thinking significantly and positively impacts academic achievement (*β* = 0.376, *p* < 0.001); critical thinking plays a partial mediating role between perceived usefulness of AI technology and academic achievement (0 is not included within the 95% confidence interval), and a full mediating role between perceived ease of use of AI technology and academic achievement (0 is not included within the 95% confidence interval).

**Discussion:**

All these results assist in more deeply comprehending learner behavior mechanisms in the era of generative AI, meanwhile contributing to theoretical support and practical insights for optimizing teaching strategies and enhancing talent development quality in universities under the context of intelligent technology. This study recommends that students use AI tools effectively while ensuring content reliability and strengthen critical thinking through questioning and practical application.

## 1 Introduction

The construction of education well guarantees the achievement of high-quality national development and national rejuvenation (Fan et al., 2024). In the existing educational system, academic performance is considered a key indicator for evaluating educational quality and effectiveness (She et al., 2025). At the same time, academic achievement, as an important personal characteristic of students, helps them gain better acceptance from peers and society (Nicolay and Huber, 2023). Since the onset of the COVID-19 pandemic in 2019, the global higher education system has undergone a dramatic transformation from traditional face-to-face teaching to technology-driven learning models (Kaur and Banga, 2025). This sudden change has not only accelerated the process of educational digitalization but has also forced university students to adapt to AI-based learning environment in a short period (Yadav, 2024). In this context, students’ acceptance of AI technology has become a crucial factor influencing their academic performance and adaptability (Xie et al., 2022).

With the release and application of AI tools of ChatGPT, Doubao, Kimi, Deepseek, and ERNIE Bot, higher education practices can no longer be discussed without considering the AI technology background (Kotsis, 2025; Xiao et al., 2025). AI exhibits excellent capabilities in information retrieval, integration, and generation, which, together with educational technologies developed over the past decades, is driving higher education into the “AI era” and into the “cyberspace” deeply empowered by AI technology (Orhan, 2022). The continuous intervention of intelligent technology presents unprecedented challenges for both university teachers and students. In this digital education environment, students perform tasks such as retrieval, conversation, and content creation by utilizing large language models, making AI a powerful assistant for personalized learning and supporting students’ autonomous learning (Wang and Li, 2024). The content generation capability of AI effectively handles repetitive tasks, making students capable of paying more attention to creative learning.

The learning challenges posed by new technologies have made education researchers realize that relying solely on teachers’ technological abilities is insufficient to address future educational transformations. Students’ understanding, attitudes, and technological literacy are equally important (Alzahrani, 2023). As AI technology gradually permeates daily learning and life, it has become an indispensable part of university students’ daily life and career preparation (Dandotiya et al., 2024). Therefore, understanding and enhancing students’ acceptance of AI technology can improve their academic performance and serve as a crucial prerequisite for cultivating talents with future competitiveness.

Critical thinking is the ability and tendency to conduct analysis and assessment as well as make rational judgments based on objective facts rather than being influenced by emotions or subjective factors (Tittle, 2011). In the context of the digital age, critical thinking is one of key high-level competencies of talents (Purwanto et al., 2023). Cultivating university students’ critical thinking abilities is a core task for universities in promoting the cultivation of innovative talents (Thornhill-Miller et al., 2023). According to the finding of Muthmainnah et al. (2022), students’ critical thinking can be positively enhanced relying on AI being applied in classroom teaching. AI-assisted teaching helps to increase students’ trust, confidence, openness, and maturity in English learning, indirectly promoting the formation of critical thinking ability. Besides, Liu and Wang (2024) stated that using AI tools in classroom teaching can effectively enhance students’ critical thinking abilities. However, the above studies are restricted to how teacher’s AI usage drives students’ cognitive abilities, but seldom highlight how students’ AI technology acceptance drives the critical thinking ability development. Furthermore, existing studies have paid little attention to the possible mediating role of critical thinking in the relationship between AI technology acceptance and academic performance.

Against this backdrop, this study aims at exploring, from the perspective of students, the influence mechanism of their perceived acceptance of AI technology on critical thinking abilities, and the mediating role of critical thinking between perceived AI technology acceptance and academic performance. This not only assists in more deeply comprehending the learner behavior mechanisms in the era of generative AI but also contributes to theoretical support and practical insights for optimizing teaching strategies and improving talent development quality in universities under the context of intelligent technology.

## 2 Literature review

### 2.1 Theoretical foundation

The Technology Acceptance Model (TAM), proposed by Davis (1989) on the basis of the Theory of Reasoned Action to examine users’ acceptance of information systems, comprises two principal determinants: (1) Perceived Usefulness (PU), which reflects the extent to which an individual believes that using a particular system will enhance their job performance; and (2) Perceived Ease of Use (PEOU), which reflects the extent to which an individual believes that using a particular system will be effortless. PU and PEU significantly predict learners’ cognitive engagement and learning involvement, both of which are positively associated with deep-learning strategies and academic achievement (Kemp et al., 2024). Based on this theoretical and empirical evidence, the present study conceptualizes PU and PEU as antecedent variables influencing whether students activate and engage in critical thinking in AI-supported learning contexts. When students perceive AI tools as both useful and easy to use, they are more likely to operate under lower levels of extraneous cognitive load and to devote their freed cognitive resources to information analysis, evidence evaluation, and reflective judgment, thereby improving their academic performance through these higher-order cognitive strategies (Kemp et al., 2024).

### 2.2 Perceived AI technology acceptance and academic performance

To explore learners’ subjective perceptions of AI technology acceptance, this study introduces two core constructs from the Technology Acceptance Model (TAM): Perceived Usefulness and Perceived Ease of Use (Davis, 1989), with the former referring to the extent to which an individual perceives that a specific technology (e.g., specific software, hardware, or system) can enhance their work efficiency or learning outcomes and the latter reflecting the individual’s perception of the effort needed for mastering and using that technology (Granić and Marangunić, 2019). Ahn (2024) stated that the ease of use of AI tools can bring into more benefits to individuals, and stimulate them to use these AI tools regularly. The utilization of the knowledge within AI tools can improve individual performance. Studies have confirmed that students can obtain advanced research, problem-solving, and learning tools from AI technology, with their academic performance improved accordingly (Crompton and Burke, 2023; Fazil et al., 2024; Ouyang et al., 2023).

In the Cognitive Load Theory, alleviating extraneous cognitive load can enhance learning outcomes (Sweller, 1988; Van Merrienboer and Sweller, 2005). Research has found that generative AI (GenAI) can enhance student learning outcomes, evidenced by increased learning speed (Möller et al., 2024), self-reported learning performance (Shahzad et al., 2025), and enhanced motivation, among other difficult-to-quantify factors (Gao et al., 2024). Therefore, generative AI (GenAI) tools can serve as cognitive aids, simplifying or scaffolding the information processing process, thus improving learning efficiency and helping students better retain information, especially when dealing with complex learning materials.

In light of this, the study raises the following three hypotheses:

*H1*: Perceived AI technology acceptance significantly and positively impacts academic achievement among Chinese undergraduates.

*H1a*: Perceived ease of use of AI technology significantly and positively impacts academic achievement among Chinese undergraduates.

*H1b*: Perceived usefulness of AI technology significantly and positively impacts academic achievement among Chinese undergraduates.

### 2.3 Perceived AI technology acceptance and critical thinking

In the case of perceiving AI technology as practically useful in daily learning and finding the process easy to use, students tend to adopt the technology (Alshammari and Babu, 2025). AI technology can help learners analyze problems more deeply, evaluate arguments, and construct logical reasoning through personalized feedback, adaptive assessments, and real-time guidance (Sajja et al., 2024). If individuals perceive these functions as useful, they tend to actively use AI tools, thereby gradually enhancing their critical thinking abilities during the interaction process (Muthmainnah et al., 2022). Furthermore, if individuals find the AI system easy to operate and the interface user-friendly, they tend to continuously use these tools for learning and reflecting, stably supporting the formation of critical thinking (Hao et al., 2024).

Perceived usefulness (PU) and perceived ease of use (PEU) have been found to significantly predict learners’ metacognitive regulation, self-directed learning strategies, and overall learning engagement, which represent higher-order learning behaviors (Michel-Villarreal et al., 2023). Learners’ subjective perceptions of the usefulness and ease of use of a given technology influence the depth of cognitive investment and the quality of information processing during learning tasks, including the use of metacognitive strategies such as planning, monitoring, and evaluation (Lin and Yu, 2023). Specifically, PU enhances learners’ perceived value and willingness to sustain engagement in learning activities, whereas PEU reduces extraneous cognitive load, thereby enabling learners to allocate cognitive resources more effectively to higher-order cognitive activities such as analysis, evaluation, and reflection (Kemp et al., 2024).

According to the finding of Zulfikasari et al. (2025), chatbot AI helps Generation Z students to cultivate critical thinking skills by helping them clarify problems, categorize information, and construct answers. Fabio et al. (2024) invited 126 participants to complete a self-assessment questionnaire to evaluate their familiarity with and experience using ChatGPT, followed by a Critical Reasoning Assessment (CRA) for the assessment of their critical thinking performance. Increasing knowledge of ChatGPT and the familiarity with it and using cautiously, can enhance critical thinking abilities. Han et al. (2025) indicated that both technology acceptance and learner engagement significantly drive critical thinking.

On these accounts, the study raises three research hypotheses:

*H2*: Perceived AI technology acceptance significantly and positively impacts critical thinking among Chinese undergraduates.

*H2a*: Perceived ease of use of AI technology significantly and positively impacts critical thinking among Chinese undergraduates.

*H2b*: Perceived usefulness of AI technology significantly and positively impacts critical thinking among Chinese undergraduates.

### 2.4 Critical thinking and academic performance

Higher-order thinking skill is the precondition of the achievement of academic goals across various disciplines (Facione and Facione, 1997). Prioritizing the development of thinking abilities, namely critical thinking, creative thinking, or problem-solving, as one of the core objectives of educational institutions, enhances students’ academic performance and helps them become more prepared and well-rounded individuals (Halpern, 2013). In the study by Pirozzi et al. (2008), individuals with high levels of critical thinking ability are capable of making effective utilization of time, cleverly planing and applying their limited time and knowledge, and effectively organizing information, problems, or decisions to ultimately draw conclusions. The most fundamental characteristic of critical thinkers is self-awareness. Individuals with critical thinking skills can well recognize their strength and weakness (Ruggerio, 1990). Moreover, critical thinkers excel in skillfully applying their existing knowledge and experience in new situations (Beyer, 1995; Ennis, 2011).

Paul and Elder (2001) defined critical thinking as the ability to analyze and evaluate individual’s thinking system, with the aim of improving one’s thinking within a set of standards defined by the individual. Students with critical thinking can critically understand their learning and thinking processes, enabling them to take on complex learning responsibilities and promoting self-monitoring. Therefore, critical thinking enables individuals to become experienced and efficient learners (Moon, 2007). By encouraging students to deeply examine, evaluate, and investigate the knowledge they acquire, critical thinking offers a higher-quality learning experience (Dwyer et al., 2014; Shehab and Nussbaum, 2015), thereby improving academic performance (Kwan and Wong, 2015). Hence, critical thinking can be considered an important skill for enhancing academic achievement (Scott and Markert, 1994). Many studies in the literature have found an obvious linkage between critical thinking skills or tendencies and academic performance (D’Alessio et al., 2019; Orhan, 2022; Rivas et al., 2023). On these accounts, this study raises the following research hypotheses:

*H3*: Critical thinking significantly and positively impacts academic achievement among Chinese undergraduates.

*H4*: Critical thinking significantly mediates the relationship between perceived AI technology acceptance and academic achievement among Chinese undergraduates.

*H4a*: Critical thinking significantly mediates the relationship between perceived ease of use of AI technology and academic achievement among Chinese undergraduates.

*H4b*: Critical thinking significantly mediates the relationship between perceived usefulness of AI technology and academic achievement among Chinese undergraduates.

## 3 Methods

### 3.1 Research design

Conducted between May and July 2025, the study involves students from four universities in China, covering the southern, northern, western, and eastern regions. The study adopted a stratified random sampling method to guarantee the representativeness of the sample. First, the participants were stratified by geographic region (southern, northern, western, eastern). Within each stratum, students were randomly selected using computer-generated random numbers to enable every student to have an equal chance of being chosen. A total of 600 students participated, with the questionnaire distributed in stages.

### 3.2 Research participants

Despite the broad application of AI tools for daily assignments among university students, educators are generally concerned that this practice may affect students’ critical thinking abilities regarding assignment topics, potentially weakening the learning outcomes students ultimately achieve. To ensure the representativeness of the sample, a stratified random sampling approach was employed. Participants were stratified according to their geographic regions (southern, northern, western, eastern), and survey data were collected from students across different types of institutions and academic majors. This study distributed 150 questionnaires in each region, enabling the sample to more accurately reflect the true characteristics of the population and thereby improving the stability and reliability of the estimates. Utilizing Chinese university students, a specific group, as study objects, this study clearly elucidates regional characteristics and provide a key pathway ensuring the proper use of AI tools in classrooms, assisting students in bettering their learning abilities. This pathway drives reform in university teaching and helps to improve students’ critical thinking ability.

The sample size should be 10 times the number of items in the questionnaire (Hair et al., 2009). The questionnaire contained 32 items, requiring a total sample size of 320. To ensure that the number of valid samples met the required quantity and that the Amos statistical analysis tool could be effectively used, 600 students were selected as participants. Interviewer distributed 600 questionnaires, with 549 returned, hence the response rate reached 91.50%, which provided a sufficient sample size. Among the participants, 341 were female (62.1%), and 208 were male (37.9%). The higher proportion of female students is consistent with the current gender ratio among Chinese university students (Xu et al., 2023).

### 3.3 Research instruments

The technology acceptance is measured using the scale developed by Davis (1989), which consists of 12 items and includes two dimensions: Perceived Ease of Use (PEU) and Perceived Usefulness (PU). The Cronbach’s Alpha values for PEU and PU are 0.942 and 0.949, respectively, with the overall AI acceptance scale at 0.951, indicating strong reliability. The AVE for AI acceptance is 0.743, exceeding 0.5 and showing good convergent validity.

The study uses the academic achievement scale of Li et al. (2016), which includes 12 items and consists of four dimensions. The Cronbach’s Alpha values for the academic achievement dimensions—interpersonal facilitation, learning cognitive ability, communication ability, and self-management—are 0.900, 0.857, 0.777, and 0.846, respectively, with the overall scale at 0.939, all >0.7, indicating strong reliability. The AVE is 0.565, exceeding 0.5 and showing good convergent validity.

Critical thinking is measured using a scale adapted by Gerlich (2025), which includes 8 items and is a unidimensional scale. Gerlich (2025) indicated that a unidimensional scale can effectively capture the overall level of the variable, providing a reliable and valid composite measure. The Cronbach’s Alpha value for the Critical Thinking scale is 0.935, indicative of a strong reliability. The AVE value for Critical Thinking is 0.645.

A Likert 5-point scoring method was used in all above scales.

### 3.4 Data gathering procedure

The distribution and collection of questionnaire were conducted on the online platform Wenjuanxing, with the assistance of university teachers who forwarded the questionnaires to students. The guidance for completing the questionnaire took approximately 3 min, and the total time for completing all three scales was about 9 min. Participants were instructed to fill out the questionnaire independently and honestly based on their personal information. In the data collection process, students were allowed to stop answering at any time, and the entire survey was conducted anonymously. It was also explained that the information collected would only be used for this study and would not be disclosed to any third parties.

### 3.5 Data analysis

The valid questionnaires collected were subjected to reliability analysis, common method bias test, descriptive analysis, correlation analysis, confirmatory factor analysis (CFA), structural equation modeling, and Bootstrap mediation effect analysis under the assistance of SPSS and AMOS.

### 3.6 Ethical considerations

This study received ethical approval from the Ethics Committee of Harbin Institute of Petroleum. All participants verbally agreed to participate in the study after fully understanding its purpose and confidentiality measures. University leadership also approved the participation of students. To ensure voluntary participation and adherence to ethical guidelines, the study strictly followed confidentiality protocols. This method aims to enhance the result generalizability and it meets advanced research standards.

## 4 Results

### 4.1 Common method bias test

To ensure that common method bias did not threaten the validity of the findings, this study adopted both procedural remedies and statistical tests. In terms of procedural control, anonymous participation was used to reduce respondents’ evaluation apprehension and thereby lower the risk of common method bias (Podsakoff et al., 2003).

For statistical diagnostics, Harman’s single-factor test was first conducted to examine potential common method variance. The exploratory factor analysis in SPSS extracted five factors with eigenvalues greater than 1, and the first factor accounted for 47.24% of the total variance, which is below the critical threshold of 50% (Hair et al., 2009).

To further detect common method bias, the variance inflation factors (VIFs) of all latent variables were calculated. The VIF values were 1.886, 1.875, and 1.461, all of which are below the recommended cutoff value of 3 (Kock, 2015), indicating the absence of substantial collinearity-based common method bias.

Taken together, these results suggest that common method bias is not a serious concern in this study.

### 4.2 Descriptive statistics and correlation analysis

Table 1 reveals a significant correlation between four dimensions. An obvious positive correlation was observed between them, as evidenced by the correlation coefficient >0. According to the correlation analysis, there are significant low and medium correlations between the variables, with no high correlations, indicating no multicollinearity issues, which allows for further analysis (Benesty et al., 2009).

**Table 1:** Descriptive statistics and Pearson correlation analysis of variables.

Variables	M	SD	Perceived usefulness	Perceived ease of use	Academic achievement	Critical thinking
Perceived usefulness	3.64	0.930	1			
Perceived ease of use	3.64	0.921	0.652**	1		
Academic achievement	3.57	0.808	0.509**	0.588**	1	
Critical thinking	3.38	0.834	0.513**	0.508**	0.607**	1

**p < 0.01.

### 4.3 Model fit analysis

For the structural validity of the research model, the absolute fit indices are *χ*^2^/df = 3.394, <the reference value of 5 (Hair et al., 2009), meeting the standard. RMSEA = 0.066, which is less than the reference value of 0.100, meeting the standard (Browne and Cudeck, 1992). GFI = 0.808, which is greater than the reference value of 0.800 (Bentler and Bonett, 1980), meeting the standard. Although the GFI is slightly below the conventional benchmark of 0.900, Bentler (1990) pointed out that the GFI value is susceptible to sample characteristics and the complexity of the structural model; therefore, its interpretation should be considered in conjunction with other indices. Relative fit indices are CFI = 0.927, IFI = 0.927, NFI = 0.900, and RFI = 0.891, all exceeding the 0.900 threshold (Abedi et al., 2015). The parsimonious fit indices are PNFI = 0.831 and PGFI = 0.856, both exceeding the 0.050 threshold (Abedi et al., 2015). Taken together, the classroom management strategy exhibits a strong model fit (Table 2).

**Table 2:** Model fit analysis.

Item	χ^2^/*df*	RMSEA	GFI	CFI	IFI	NFI	RFI	PNFI	PGFI
Value	3.394	0.066	0.808	0.927	0.927	0.900	0.891	0.831	0.856

### 4.4 Path analysis of the model

In the structural equation model, the path coefficient size represents the magnitude of influence of the independent variable on the dependent variable, and the effect size can be interpreted as the sum of direct and indirect effects. Perceived usefulness of AI technology and perceived ease of use of AI technology significantly and positively impact academic achievement (*β* = 0.117, *t* = 2.413, *p* < 0.050; *β* = 0.363, *t* = 7.338, *p* < 0.001), and critical thinking (*β* = 0.332, *t* = 6.054, *p* < 0.001; *β* = 0.313, *t* = 5.703, *p* < 0.001); critical thinking significantly and positively impacts academic achievement (*β* = 0.376, *t* = 8.904, *p* < 0.001) (Table 3).

**Table 3:** Path analysis of hypotheses.

Hypothesis path	Path coefficient	t-value
Perceived Usefulness of AI Technology → Academic Achievement	0.117*	2.413
Perceived Ease of Use of AI Technology → Academic Achievement	0.363***	7.338
Perceived Usefulness of AI Technology → Critical Thinking	0.332***	6.054
Perceived Ease of Use of AI Technology → Critical Thinking	0.313***	5.703
Critical Thinking → Academic Achievement	0.376***	8.904

***p < 0.001, *p < 0.05.

### 4.5 Mediation effect and bootstrap analysis

In structural equation modeling, mediation effects are tested by checking whether the effect does not include 0 within the confidence interval (CI) MacKinnon and Luecken (2008). The mediation effect does not include 0 at 95% CI, i.e., *p* < 0.05 (Cheung and Lau, 2008; Lau and Cheung, 2012). Therefore, this study follows the standard of Bootstrap mediation effect structural equation modeling analysis to analyze indirect effects. Repeated sampling of 2,000 times is required to calculate the CI (Tibshirani and Efron, 1993). The indirect effect does not include 0 within the 95% CI, reaching significant level (MacKinnon and Luecken, 2008). Both the direct and indirect effects do not include 0 within the 95% CI, and the total effect does not include 0 within the 95% CI, both reaching significant level, indicating partial mediation; the direct effect includes 0 within the 95% CI and is not significant, but both the indirect effect and total effect do not include 0 within the 95% CI and reach a significant level, hinting full mediation. According to the analysis results (Table 4), critical thinking plays a full mediation role between perceived usefulness of AI technology and academic achievement; and a partial mediation role between perceived ease of use of AI technology and academic achievement.

**Table 4:** Mediation effect and bootstrap analysis.

Hypothesis path	Direct effects	Indirect effects	Total effects
Perceived Usefulness of AI Technology → Academic Achievement	[−0.050, 0.242]	[0.070, 0.189]	[0.111, 0.360]
Perceived Ease of Use of AI Technology → Academic Achievement	[0.248, 0.484]	[0.075, 0.177]	[0.364, 0.606]
Perceived Usefulness of AI Technology → Critical Thinking	[0.204, 0.446]		[0.204, 0.446]
Perceived Ease of Use of AI Technology → Critical Thinking	[0.206, 0.427]		[0.206, 0.427]
Critical Thinking → Academic Achievement	[0.274, 0.471]		[0.274, 0.471]

## 5 Discussion

The study specifically focuses on the relationships between students’ perceived AI technology acceptance, critical thinking abilities, and academic achievement. According to the model analysis results, perceived AI technology acceptance and critical thinking significantly determine students’ academic achievement. The finding assists in well comprehending the learning process when students use AI tools.

### 5.1 Perceived AI technology acceptance and academic achievement

Students’ perceived AI technology acceptance can significantly and positively impact academic achievement, and the finding conforms to other studies (Navarro et al., 2023). Regarding perceived usefulness, Chinese university undergraduates have clear learning goals for using AI technology tools, which allows them more flexibility in selecting learning content that fits their personal learning characteristics. This also reflects more personalized feedback and real-time guidance, giving students better control over their learning content and pace. By using AI tools, students can broaden their perspectives, achieve their learning goals, and more deeply understand knowledge, thereby making their overall competency and learning abilities well elevated. Regarding perceived ease of use, Chinese undergraduates are familiar with the operational processes of AI tools and find their interfaces more intuitive and their operations easier. This makes students more willing to use AI tools for information retrieval, data analysis, and knowledge construction, etc. during their studies. This process reduces students’ cognitive load, making them capable of focusing more on understanding and applying knowledge, which positively affects their academic achievement.

### 5.2 Perceived AI technology acceptance and critical thinking

Both perceived AI technology acceptance can significantly and positively impact critical thinking, conforming to other studies (Muthmainnah et al., 2022). In higher education, AI helps to reshape critical thinking and educational practices. The advocacy for adjusting learning methods, emphasizing higher-order thinking and real-world application, makes AI an enabler of critical thinking (Smolansky et al., 2023). Recent studies have explored how perceived usefulness of AI affects critical thinking skills in education. AI-based teaching, based on perceived usefulness, can positively impact students’ trust, self-confidence, and openness, accordingly enhancing students’ critical thinking skills. According to existing studies, perceived ease of use of AI technology can strengthen university students’ critical thinking abilities, with 64% of respondents believing that AI technology tools greatly improved their critical thinking abilities (Ruiz-Rojas et al., 2024). It can supplement the learning process, well benefiting the cultivation of critical thinking ability (Rusandi et al., 2023).

### 5.3 Critical thinking and academic achievement

In our study, critical thinking is found to significantly and positively affect students’ academic achievement. This result conforms to other studies (Saido et al., 2017). Critical thinking is a cognitive ability aimed at enhancing higher-order cognition and intellectual capacity. Skills such as analysis, deduction, and reasoning within critical thinking can improve cognitive abilities, organizing and understanding reasoning, interpretation, and more (Wicaksana et al., 2020). Ghanizadeh (2017) found in a survey of university students that critical thinking facilitates the absorption and understanding of knowledge, impacting academic achievement. In higher education settings, teachers encourage students to improve critical thinking through cognitive training during the teaching process (Karbalaei, 2012). Engaging students’ enthusiasm and training critical thinking during learning promotes academic progress. Research also shows that learners with high critical thinking abilities attempt to comprehend and think critically about what they are learning, and make critical analysis and evaluation. Critical thinking can improve students’ cognitive processes, which is crucial for personal success, thus enhancing students’ academic achievement (Orhan, 2022).

### 5.4 Mediating role of critical thinking

In our study, critical thinking can mediate the relationship between AI technology acceptance and academic achievement, which conforms to other studies (Winne and Azevedo, 2022). When perceiving AI tools as useful and able to effectively improve learning efficiency and outcomes, students are inclined to invest time and effort, resulting in better cognitive depth and persistence in their learning. Perceived usefulness enhances students’ learning confidence and promotes deeper knowledge processing and positive learning behaviors (Müller and Goldenberg, 2021). In the case of perceiving AI tools as easy to use, with simple operations, user-friendly interfaces, and low learning costs, students tend to use these tools frequently and maintain continuous engagement, accordingly strengthening the learning efficiency and depth of knowledge acquisition. Perceived ease of use directly promotes learning behaviors and indirectly impact academic achievement by enhancing critical thinking abilities. Perceived ease of use boosts students’ sense of competence and learning confidence, which in turn drives deeper knowledge processing and positive learning behaviors. Critical thinking helps students more effectively discern and utilize the information provided by AI tools while maintaining independent thinking and reflection during knowledge construction and problem-solving, ultimately leading to improved academic achievement.

The unique contribution of this study lies in revealing the mediating role of critical thinking between AI technology acceptance and academic achievement. The findings indicate that critical thinking fully mediates the relationship between perceived usefulness of AI technology and academic achievement, highlighting the importance of critical thinking as a cognitive mechanism.

## 6 Conclusion

### 6.1 Contributions

In higher education, AI tools are reshaping educational practices. Critical thinking is regarded as a vital ability for strengthening students’ higher-order thinking skill. Despite the previous finding regarding the positive impact of teacher use of AI on students’ critical thinking, how AI tools drive academic achievement among undergraduate students is scarcely examined. Against this backdrop, we proposed research hypotheses and built a mediation model for examining the relevance of students’ perceived AI technology acceptance to academic achievement. Through a questionnaire survey, this study found that perceived AI technology acceptance and critical thinking can significantly and positively impact academic achievement, separately. Furthermore, critical thinking mediates the relationship between perceived AI technology acceptance and academic achievement. This study more deeply elucidates the relationship between perceived AI technology acceptance and academic achievement, highlighting the critical role of critical thinking. Therefore, this study enriches research on AI technology acceptance and academic achievement and assists in merging AI in higher education management in practice.

### 6.2 Recommendations

Academic Applications of AI Tools: To fully realize the value of AI tools in students’ learning processes, students can apply AI tools for personalized learning to address knowledge gaps and improve learning efficiency. Additionally, AI tools should be applied in subject-specific projects like data analysis and generation. Finally, upon the application of AI, the reliability and accuracy of the generated and analyzed content shall be guaranteed, avoiding blind adoption of generated results, and promoting high-quality, sustainable self-directed learning.

Enhancing Students’ Critical Thinking Ability: To enable AI tools to the most effectively enhance students’ critical thinking ability, students should develop the habit of questioning the output of AI tools, cross-checking results, and conducting logical analysis to prevent blind trust in results, which could lead to errors. Moreover, the interactive functions of AI tools should be used to foster independent thinking. Lastly, integrating AI tools into subject research and practical teaching projects can help students design problems, analyze data, and continue to enhance their critical thinking throughout the learning process.

### 6.3 Research limitations and future research directions

This study used a unidimensional scale to measure critical thinking, which is incapable of fully reflecting the multidimensionality and complexity of critical thinking. Future research could adopt multidimensional or domain-specific measurement tools to elucidate the mediating roles of various critical thinking dimensions. Secondly, the measurement of academic achievement relied on self-reports, hence could be influenced by subjective bias and social desirability effects. Future research could combine teacher evaluations or objective academic performance records to improve the accuracy and reliability of academic achievement measurements. Lastly, the sample in this study exhibited imbalanced gender distribution, with 62.1% female and 37.9% male participants. This imbalance primarily arose because the participating universities were liberal arts and teacher training institutions, where the proportion of female students is typically higher. Future studies should include more balanced samples across different types of universities and disciplines to make the research findings more generalized.

## Data Availability

The raw data supporting the conclusions of this article will be made available by the authors, without undue reservation.
